# Disseminating an Elder Mistreatment Electronic Health Record Tool in a National Health System

**DOI:** 10.1093/geroni/igaf122.091

**Published:** 2025-12-31

**Authors:** Lena Makaroun, Colleen McQuown, Luna Ragsdale

**Affiliations:** VA Pittsburgh Healthcare System/University of Pittsburgh, Pittsburgh, Pennsylvania, United States; Louis Stokes Cleveland VA Medical Center, Cleveland, Ohio, United States; Durham VA Healthcare System, Durham, North Carolina, United States

## Abstract

The Veterans Health Administration (VHA), the largest integrated health system in the United States, provides comprehensive, high-quality care to approximately 5 million Veterans age ≥60 years, many with a high burden of elder abuse (EA) risk factors. VHA is a leader in research and care for complex geriatric syndromes and integrating social and medical care, however recent reviews identified a need for systems support for addressing EA. Through interdisciplinary collaborations between VHA researchers and clinical leaders in geriatrics and emergency medicine, we developed a new electronic health record (EHR) tool to support standardized a clinical process for EA identification. First, we created a national evidence-based EHR EA screening template by adapting the Elder Mistreatment Screening and Response Tool (EM-SART) to the VHA system and workflow. Second, we obtained broad stakeholder feedback on the note from leaders of different clinical service lines. Finally, the note template was piloted at three sites in preparation for dissemination. The adapted EM-SART was released for national dissemination in February 2025. In this symposium, we present practical experiences, empirical data and lessons learned from stakeholder engagement, piloting, refinement, implementation and national dissemination of an adapted tool for improving EA identification and response in a national healthcare system. Highlighting how VHA has leveraged partnerships between researchers, philanthropic foundations, and clinical operations partners to advance care for the high priority, but previously under-addressed, issue of EA can help advance implementation science knowledge for other systems looking to advance clinical care and data standardization around EA in their patients.

